# Limited Knee-Joint Range of Motion in Patients With Tophaceous Gout Improved With Medical Treatment: A 18-Months Follow Up

**DOI:** 10.3389/fmed.2020.00074

**Published:** 2020-02-28

**Authors:** Chuan-Chin Lu, James Cheng-Chung Wei, Cheng-Ang Chang, Chih-Ming Chen, Sen-Wei Tsai, Chih-Jung Yeh

**Affiliations:** ^1^Department of Rheumatology, Physical Medicine and Rehabilitation, Taichung Tzu Chi Hospital, Buddhist Tzu Chi Medical Foundation, Taichung, Taiwan; ^2^Department of Physical Therapy, Hung Kuang University, Taichung, Taiwan; ^3^Division of Allergy, Immunology and Rheumatology, Chung Shan Medical University Hospital, Taichung, Taiwan; ^4^Graduate Institute of Integrated Medicine, China Medical University, Taichung, Taiwan; ^5^Department of Radiology, Taichung Hospital, Ministry of Health and Welfare, Taichung, Taiwan; ^6^School of Public Health, Chung Shan Medical University, Taichung, Taiwan; ^7^Education and Research on Geriatrics and Gerontology, Chung Shan Medical University, Taichung, Taiwan

**Keywords:** gout, tophi, intra-articular, magnetic resonance imaging, urate-lowering therapy

## Abstract

**Objectives:** Tophi may occur within the knee joint causing limited knee-joint range of motion (knee motion). We investigated the relationships between knee motion, total intra-articular tophi size (tIA-tophi), and total subcutaneous tophi size (tSC-tophi) and determined knee motion improvement after continual urate-lowering therapy (ULT).

**Methods:** A total of 26 patients with tophaceous gout and limited knee motion were enrolled. Inclusion criteria were age ≤ 60 years and preserved knee joint space on a plain radiograph. tSC-tophi were measured using a Vernier caliper and tIA-tophi were measured using magnetic resonance imaging software. All patients were re-evaluated after 12–24 months of continual ULT. Upon initial visit, knee motion was related to tIA-tophi and tSC-tophi.

**Results:** After an average of 18.2 months of ULT, the reduction in tSC-tophi was correlated with the reduction in tIA-tophi (*P* = 0.014, *r* = 0.395) and improvement in knee motion (*P* = 0.038, *r* = 0.408). Knee motion can be eventually improved even in cases of poor initial knee motion (*P* = 0.000, *r* = –0.911) or large initial tIA-tophi (*P* = 0.014, *r* = 0.476). Tophi can occur in any location within the knee joint. In multiple lineal regression, knee motion was predicted to improve 8.39° for every 10cm of tIA-tophi reduction.

**Conclusions:** Limited knee motion in patients with intra-articular tophi improved with medical treatment, regardless of initial severity. Furthermore, tSC-tophi can be used as an indicator of limited knee motion and their improvement as a predictor of knee motion improvement.

## Introduction

Tophi typically occur many years after uncontrolled gouty arthritis and present in subcutaneous tissue (1). It can, however, occur in the absence of previous gout (2) and in unusual sites such as bones, tendons, and joints.

Patients with gout have an increased prevalence and severity of knee osteoarthritis (3). Tophi occurring in knee joints can lead to limited knee-joint range of motion (knee motion) (4–6), resulting in knee deformity and walking difficulties and often been regarded as being due to knee osteoarthritis. Patients with gout and limited knee motion were unexpectedly found to have intra-articular tophi through magnetic resonance imaging (MRI) (4) and arthroscopy (5). The treatment approach in such cases has been arthroscopic removal of the intra-articular tophi (5, 7), even with total knee arthroplasty (8, 9). Total knee arthroplasty in these patients may have higher possibility of poor wound healing and other complication, needing revision surgery (10). A study reported that painful knee locking caused by intra-articular tophi was successfully treated through allopurinol therapy in a 67-year-old man (6). However, further investigation is required to determine if urate-lowering therapy (ULT) is a favorable alternative to arthroscopic removal.

Whether knee motion limitation is correlated with total intra-articular tophi size (tIA-tophi) and total subcutaneous tophi size (tSC-tophi) has not been determined. Studies have noted that subcutaneous tophi could be successfully reduced with ULT (11, 12). Whether successful reduction of subcutaneous tophi with continual ULT is correlated with a reduction of intra-articular tophi and hence improves knee motion remains unclear; few studies have presented this correlation. We evaluated the hypothesis that tIA-tophi and tSC-tophi could be simultaneously regressed through continual ULT and that any related knee motion limitations could be improved, and therefore speculate that such patients might be treated well with medical rather than surgical treatment.

## Materials and Methods

### Patients

From January 2010 to January 2015, we recruited and conducted face-to-face interviews in a medical rheumatologic center with patients with tophaceous gout and limited knee motion. All the patients fulfilled the 1977 American Rheumatism Association criteria for gout (13). We considered a patient as having gout only if he or she had had an acute episode that was so severe that it caused difficulty in walking or using the affected joint; had an episode that was most painful within 24 h and was resolved within 14 days; and had no pain between symptomatic episodes. These criteria also fulfill the 2015 gout classification criteria of the American College of Rheumatology (ACR) and the European League against Rheumatism (EULAR) (14). A subcutaneous tophus, defined as a draining or chalk-like subcutaneous nodule under the skin, often with overlying vascularity, is also a criterion in the 2015 criteria (14).

We included patients with tophaceous gout and limited knee motion, age ≤ 60 years, and a healthy knee-joint space on a plain radiograph. Accordingly, data of 30 patients were collected in this study. MRI (Siemans, Erlangen, Germany) was conducted on the right or left knee, according to which had more limited motion.

Clinical characteristics were recorded during the initial visit, including sex, age at visit, age at gout and subcutaneous tophi onset, subcutaneous tophi duration, knee motion, tSC-tophi, and tIA-tophi.

**Figure 1:**
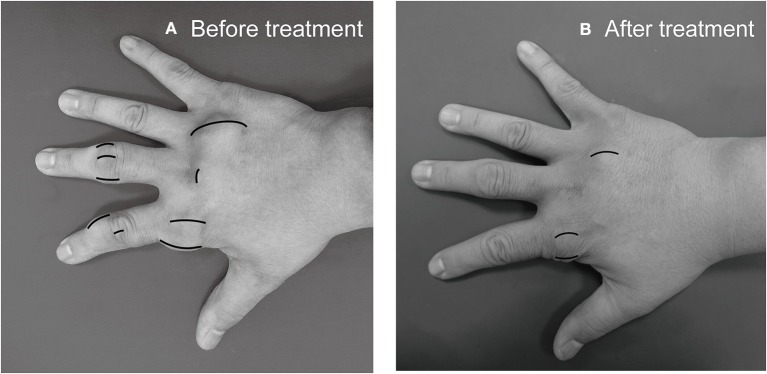
Subcutaneous tophi evaluation and response assessment. **(A)** Baseline subcutaneous tophi on the right hand of a 31-year-old man with tophaceous gout exhibiting multiple subcutaneous tophi. A visible subcutaneous tophus was measured according to its longest diameter by using a Vernier caliper. **(B)** Follow-up measurement revealed decreased subcutaneous tophi size.

**Figure 2:**
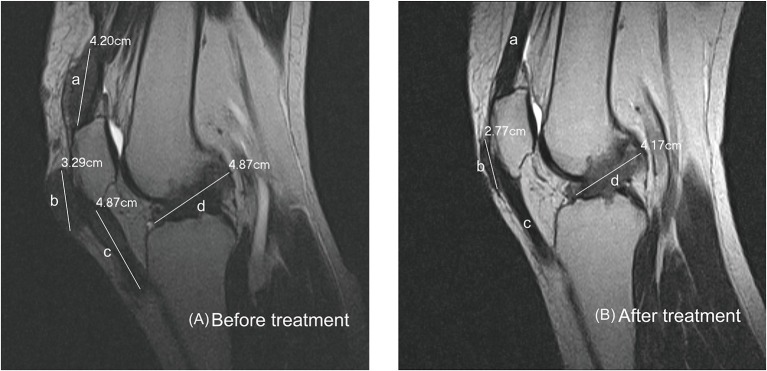
Intra-articular tophi evaluation and response assessment. **(A)** Baseline MRI of the knee joint of a 31-year-old man with subcutaneous tophaceous gout revealed multiple intra-articular tophi. A tophus within the knee joint was measured according to response evaluation criteria in solid tumors criteria. The total length in this plane is the sum of *a* + *b* + *c* + *d*, 17.23 cm. **(B)** Follow-up MRI revealed decreased intra-articular tophi size. The tophi at *a* and *c* dissolved, and those at *b* and *d* decreased.

Subcutanous tophi duration, measured in years, was determined as the time period from subcutaneous tophus onset to initial visit. A visible subcutaneous tophus was measured according to its longest diameter using a Vernier caliper (11) (Figure 1); this approach was endorsed as an index tophus measurement in the outcome of a rheumatology clinical trial (15). Intra-articular tophus was assessed by a radiologist, who did not know the clinical information and disease severity of the enrolled patients. A tophus within the knee joint was measured according to Response Evaluation Criteria in Solid Tumors (RECIST) criteria (16) (Figure 2), in which unidimensional measurement of the longest diameter was used for all measurable tumor, using MRI computer software (Syngo Multimodality Workplace, Siemens, Erlangen, Germany); this approach was widely used as therapeutic effect for tumor size reduction in oncology so far (17). tSC-tophi and tIA-tophi, recorded in centimeters, were determined as the sum of the diameters of all measurable tophi over subcutaneous tissue and within the knee joint, respectively. Knee motion, measured in degrees using a goniometer, was determined as the angle between complete knee extension and maximum knee flexion.

In each patient, combined allopurinol and benzbromazone therapy was prescribed according to 2006 EULAR evidence-based recommendations for gout (18). Allopurinol began at a low dose (50–100 mg daily) and was increased by 100 mg every 4 weeks. Both drugs were titrated upwards to achieve a serum-urate level of <5.0 mg/dL, which fulfilled the 2012 ACR guidelines (19) and 2016 updated EULAR (20) recommendations for the management of gout. During the follow-up period, each patient continued to use the dose that was adjusted according to the target serum-urate level.

A total of 26 patients who completed continual ULT, repeated tSC-tophi measurement, repeated knee-motion measurement, and MRI evaluation during follow-ups of 12 to 24 months were enrolled in this study. Of these patients, 16 (61.5%) did not have visible subcutaneous tophi over the knee joints on physical examination at the first evaluation. They were all diagnosed as osteoarthritis and ever suggested surgical removal of tophi or total knee arthroplasty by orthopedists. The study was approved by the Institutional Review Board of Taichung Hospital, Taiwan.

### Statistical Analysis

All analyses were performed using SPSS Statistics, Version 19 for Windows 7.0. Statistical significance was defined as a two-sided *P*-value < 0.05. Continuous data were compared through a paired *t-*test and independent *t-*test. Pearson correlation was used to test the relationships between knee motion, tSC-tophi, and tIA-tophi, before and after continual ULT. Multiple linear regression was used to verify the hypothesis that tIA-tophi reduction was a crucial factor in knee-motion improvement.

## Results

### Baseline Clinical Characteristics

Clinical characteristics of the 26 enrolled patients are summarized in Table 1. All the patients were male and had intra-articular tophi of the knee on MRI. Table 1 shows that the patients' tSC-tophi, tIA-tophi, and knee motion were all improved after continual therapy (all *P* = 0.000).

**Table 1:** Clinical features of 26 patients with tophaceous gout and limited knee-joint range of motion.

**Characteristics**	**Before ULT**	**After ULT**	***P*****-value**	
			**Paired *t*-test**	***t*-test**
Male sex, *n* (%)	26 (100%)			
Presence of intra-articular tophi, *n* (%)	26 (100%)			
Subcutaneous tophi over knee, *n* (%)	16 (61.5%)			
Age at initial visit (yr)	48.4 ± 9.8			
Age at gout onset (yr)	33.2 ± 10.2			
Age of tophi onset (yr)	40.3 ± 9.9			
Subcutaneous tophi duration (yr)	8.7 ± 6.2			
Difficulty in squatting (yr), *n* = 23	4.7 ± 3.7			
Total subcutaneous tophi (cm)	52.8 ± 40.5	8.1 ± 12.5	<0.001	<0.001
Total intra-articular tophi (cm)	18.0 ± 9.5	8.2 ± 4.7	<0.001	<0.001
Knee joint range of motion (degree)	103.5 ± 5.7	129.6 ± 6.5	<0.001	<0.001

*Comparison before and 18.2 months (standard deviation [SD]: 5.9 months) after urate-lowering therapy (ULT)*.

*Data are expressed as mean ± standard deviation or number (percentage), p < 0.05 considered significant*.

### At Initial Visit

**Figure 3:**
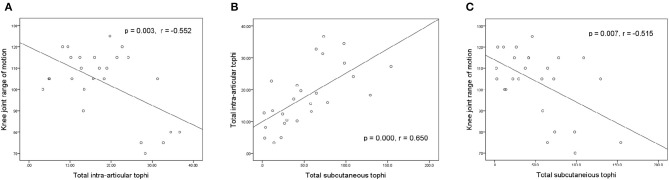
Relationships between knee-joint range of motion and tophi size at initial visit. **(A)** Knee-joint range of motion vs. total intra-articular knee tophi size; **(B)** total intra-articular vs. total subcutaneous tophi size; **(C)** knee-joint range of motion vs. total subcutaneous tophi size.

Figure 3 illustrates the relationships between knee motion, tIA-tophi, and tSC-tophi at the patients' initial visit. The degree of knee motion is inversely related to tIA-tophi in the knee in Figure 3A (*P* = 0.003, *r* = −0.552). tIA-tophi is almost linearly related to tSC-tophi in Figure 3B (*P* = 0.000, *r* = 0.650). Additionally, a negative relationship is illustrated between the degree of knee motion and tSC-tophi in Figure 1C (*P* = 0.007, *r* = –0.515).

### After Treatment

**Figure 4:**
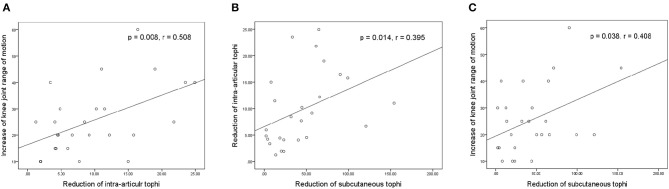
Relationships between knee-joint range of motion and tophi size after continual urate-lowering therapy. **(A)** Increase in knee-joint range of motion vs. reduction in total intra-articular knee tophi size; **(B)** reduction in total intra-articular vs. total subcutaneous tophi size; **(C)** increase in knee-joint range of motion vs. reduction in total subcutaneous tophi size.

Figure 4 presents the relationships between the increase in knee motion and reductions in tIA-tophi and tSC-tophi after an average of 18.2 months of continual ULT. In Figure 4A, the increase in knee motion is strongly related to the reduction in tIA-tophi (*P* = 0.008, *r* = 0.508). In Figure 4B, the reduction in tIA-tophi is associated with that in tSC-tophi (*P* = 0.014, *r* = 0.395). Moreover, Figure 4C shows that the increase in knee motion is also related to the reduction in tSC-tophi (*P* = 0.038, *r* = 0.408).

**Figure 5:**
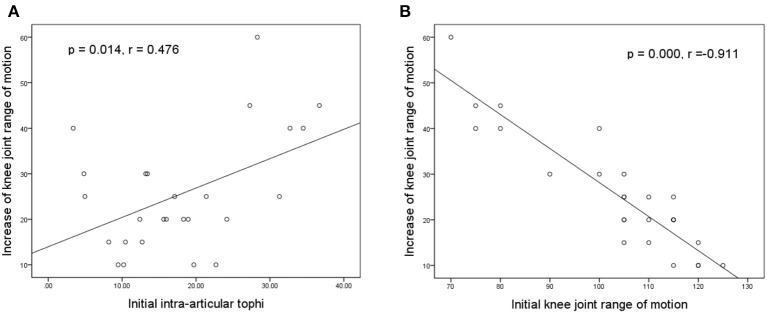
Relationships between the increase in knee motion and the initial knee variables. Increase in knee-joint range of motion vs. **(A)** initial total intra-articular tophi size and **(B)** initial knee-joint range of motion.

Figures 5A,B illustrate the association of the final increase in knee motion with initial tIA-tophi and initial knee motion, respectively. Increase in knee motion was associated with initial tIA-tophi (Figure 5A; *P* = 0.014, *r* = 0.476) and strongly associated with initial knee motion (Figure 5B; *P* = 0.000, *r* = –0.911).

### Tophi Site Within the Knee Joint

Table 2 shows the intra-articular tophi sites in knee joints on MRI. Tophi can occur in any location within the knee joint, with the three most common sites in this study being the lateral rim of the lateral femoral condyle (92.3%), intercondylar fossae (84.6%), and infrapatellar fad and anterior joint recess (76.9%). A total of 23 patients (88.5%) had tophi at three or more sites. Additionally, 13 patients (50.0%) had tophi at five or more sites.

**Table 2:** Intra-articular tophi sites and total number of tophi sites within the knee joint.

**MRI findings**	***n* (%)**
Intra-articular tophi site	
Infrapatellar fad & anterior joint recess	20 (76.9%)
Intercondylar fossae	22 (84.6%)
Tibial eminence	4 (15.4%)
Lateral rim of lateral femoral condyle	24 (92.3%)
Lateral rim of tibial plateau	15 (60.0%)
Medial rim of medial femoral condyle	16 (61.5%)
Medial rim of tibial plateau	15 (60.6%)
Total number of tophi sites	
≥ 2	25 (96.2%)
≥ 3	23 (88.5%)
≥ 4	18 (69.2%)
≥ 5	13 (50.0%)
≥ 6	8 (30.8%)

*Magnetic resonance imaging (MRI) was employed to identify tophi sites in 26 patients*.

*Data are expressed as number (percentage)*.

### Determinants of Knee-Motion Improvement

Table 3 presents linear regression models with increase in knee motion as the dependent variable and reduction of tIA-tophi as the independent variable, with three covariables: age at initial visit, subcutaneous tophi duration, and presence of visible subcutaneous tophi over the knee joint. The reduction of tIA-tophi was discovered to be a significant variable affecting the improvement in knee motion (Model 1; *P* = 0.013), and this finding remained significant after adjusting for all additional variables (Model 4; *P* = 0.019). By contrast, in Model 4, age at initial visit, subcutaneous tophi duration, and presence of extra-articular tophi over the knee were all non-significant variables (*P* = 0.644, 0.942, and 0.622, respectively).

**Table 3:** Multiple linear regression analysis to determine factors for improving knee-joint range of motion.

	**Model 1**		**Model 2**		**Model 3**		**Model 4**	
	**Coefficient β**	***P***	**Coefficient β**	***P***	**Coefficient β**	***P***	**Coefficient β**	***P***
Reduction of tIA-tophi	0.769	0.013	0.783	0.013	0.781	0.017	0.839	0.019
Age at initial visit			−0.126	0.557	−0.124	0.588	−0.109	0.644
Subcutaneous tophi duration					−0.013	0.971	0.027	0.942
Extra-articular knee tophi							−2.334	0.622

*Dependent variable: improvement of knee-joint range of motion; Independent variables: Model 1, reduction of total intra-articular tophi size (tIA-tophi); Model 2, reduction of tIA-tophi and age at initial visit; Model 3, reduction of IA-tophi, age at initial visit, and subcutaneous tophi duration; Model 4, reduction of IA-tophi, age at initial visit, subcutaneous tophi duration, and presence of extra-articular knee tophi*.

## Discussion

Our study demonstrated that all our 26 patients have reduction in tSC-tophi and simultaneous improvement in knee motion after 18.2 months of continual ULT, regardless of initial severity. Additionally, tSC-tophi can be used as a useful indicator and predictor in assessing the severity and predicting the potential improvement in knee motion.

At the patients' initial visit, the severity of knee-motion limitation was strongly associated with larger tIA-tophi, which was also closely associated with larger tSC-tophi. In addition, the severity of knee-motion limitation was strongly associated with larger tSC-tophi. The current findings suggest that the larger tSC-tophi and tIA-tophi, the more limited the patient's knee motion was.

After continual ULT, tSC- and tIA-tophi were lower and knee motion was larger. The increase in knee motion was related to the decrease in tIA-tophi, which was also significantly related to the decrease in tSC-tophi. Additionally, the increase in knee motion was significantly associated with the decrease in tSC-tophi. These findings suggest that when subcutaneous tophi were successfully treated with ULT, knee motion was improved. Therefore, we can predict the improvement in knee motion when tSC-tophi decreases progressively.

Patients with larger tIA-tophi or tSC-tophi or with more limited knee motion are presumed to have a more serious condition. However, our study revealed that the magnitude of knee motion improvement was larger in those whose initial knee motion was smaller or when initial tIA-tophi was larger. A possible cause is the ceiling phenomenon in the patients with more initial knee motion or smaller initial tIA-tophi. Nevertheless, this finding suggests that room for improvement exists in patients who have poorer initial knee motion or larger initial tIA-tophi. Therefore, the clinical implication is that such a limitation can be improved regardless of how limited the initial knee motion or large the initial tIA-tophi is.

Subcutaneous tophi occur in patients with a long duration of gout and high serum urate (1). Moreover, the presence of intra-articular tophi is related to the long gout duration (21). Studies have reported that intra-articular tophi are strongly associated with bone erosion revealed using MRI (22, 23). Another study reported that intra-articular tophi could destroy the knee structure and decrease knee motion (24). Unexpectedly, in our study, tIA-tophi and severity of knee-motion limitation were not related to subcutaneous tophi duration (not shown in the figures). Our patients presented similar bone erosion by tophi on MRI scans, but all had improvement in knee motion after continual ULT. A possible cause is that our enrolled patients had preserved knee-joint space before treatment; that is, they began to receive ULT before irreversible knee-joint destruction. This study suggests that these patients should be treated early so as not to permanently damage their joint.

Tophi can be found anywhere within the knee joint (Figure 2), resulting in various degrees of knee-motion limitation. The three most common tophus sites in our study were similar to those in a study regarding the MRI patterns of 30 patients with tophaceous gout (22). In our study, 88.5% of patients presented tophi at three or more sites. In addition, 84.6% of our patients exhibited tophi in the intercondylar fossa, some of which was deposited around the anterior or posterior cruciate ligaments. For these reasons, tophi within knee joints are difficult to remove completely through surgery. Conversely, this study shows that all tophi can be diminished through continual ULT. This finding crucially reveals that intra-articular tophi require medical rather than surgical treatment.

As shown in Table 3, the principle determinant of knee-motion improvement was reduction in tIA-tophi. By contrast, subcutaneous tophi duration was not a significant variable affecting knee-motion improvement, and neither was the presence of extra-articular tophi in the knee. In linear regression analysis, knee motion increased by 8.39° for every 10 cm of tIA-tophi reduction. In our patients, tSC-tophi reduced from 52.8 to 8.1 cm, and knee motion increased eventually from 103.5 to 129.6° after continual therapy (Table 1).

MRI has a low to intermediate and variable signal intensity on T1- and T2-weighted images, respectively (22, 23), allowing early detection of gouty tophi (22). MRI can be used to visualize deeper structure and is useful to identify tophi deposition in the knee joint (4, 25, 26), which is useful to repeat assessment, and to monitor both extent and progression of disease (27). We used MRI to assess tophi response to ULT according to RECIST guideline. Just as assessment of tumor progression (28), our study revealed that MRI can be used for assessment of a decrease in tIA-tophi. Indeed, tophus is usually amorphous shape and not a straight linear, but there is no better way to describe the tophi size and its reduction so far.

Tophi are usually located in subcutaneous tissue and can be easily diagnosed without imaging evaluation. In our study, initial tSC-tophi was strongly related to initial tIA-tophi. Moreover, after treatment, the reduction in tSC-tophi was correlated with that in tIA-tophi. Thus, tSC-tophi and tIA-tophi both existed and after ULT, decreased simultaneously, which reflected that the tophi deposited within the joint were actually part of those in the whole body. However, MRI is not suitable as a routine clinical tool for assessment of tophaceous gout due to its high cost. Therefore, tSC-tophi evaluation can play a crucial role to replace MRI assessment of tIA-tophi as an indicator of limitation of initial knee motion, as well as a predictor of improvement in tIA-tophi and knee motion.

The strength of this longitudinal design study is the observation that simple drug treatment and its accompaniment through the decrease in number and size of subcutaneous tophi with multimodality assessment is sufficient to predict the improvement in the range of articular movement. Moreover, this study provided a recommendation that such involvement can be improved after urate-lowering therapy, leading to potentially preventable total joint replacement.

There are some limitations to the present study. First, Such severe involvement is not common in clinical practice; and furthermore, such patients were diagnosed as having osteoarthritis that caused their knee-motion limitation so that they did not visit rheumatologist until their subcutaneous tophi enlarged, thus limiting our sample size. Second, the knee motion limitation started on average 3.4 years after the development of subcutaneous tophi and got worse from that time. Moreover, the knee-motion limitation of all our patients was bilateral and improved after medical treatment. Therefore, their knee-motion limitation is most likely due to intra-articular tophi assessed by MRI. Nevertheless, a patient with chronic tophaceous gout usually represents that he did not receive appropriate treatment before visiting our hospital. Any patient with recurrent gout attacks at knee joint without appropriate treatment might increase the likelihood and thus might be as a mediator of motion limitation. Third, we used the total length of the tophi to represent the total size of the tophi, which may be biased.

## Conclusion

Patients with gout may have intra-articular tophi causing movement limitations. Twenty six patients with tophacous gout and knee-motion restriction were analyzed and had total intra-articular and subcutaneous tophi size measured as well as the amplitude of knee movements before and after urate-lowering therapy. After 12 to 24 months of treatment, there was an increase in the range of motion in the knees which correlated with reduction in total subcutaneous tophi size and with that in total intra-articular tophi size despite initial severity. Additionally, total subcutaneous tophi size can be used as an indicator of limited knee motion and their improvement as a predictor of knee motion improvement. Physician should be aware that knee motion limitation may be related to intra-articular tophi even without any visible tophi on the knee surface and that medical treatment can yield favorable clinical outcome.

## Data Availability Statement

The datasets generated for this study are available on request to the corresponding author.

## Ethics Statement

The studies involving human participants were reviewed and approved by Institutional Review Board of Taichung Hospital. The patients/participants provided their written informed consent to participate in this study.

## Author Contributions

C-CL, C-AC, and JW designed, acquired, and analyzed data. JW, C-JY, C-MC, and S-WT interpreted data and revised the draft manuscript. All authors reviewed and approved the final version of the manuscript.

### Conflict of Interest

The authors declare that the research was conducted in the absence of any commercial or financial relationships that could be construed as a potential conflict of interest.
